# Defective neuronal and oligodendroglial differentiation by FTD3- and ALS17-associated Ile29-to-Val mutation of CHMP2B

**DOI:** 10.1016/j.ymgmr.2019.100458

**Published:** 2019-02-02

**Authors:** Minami Yamawaki, Masumi Akiba, Naoto Matsumoto, Natsumi Watanabe, Kohei Hattori, Yu Takeuchi, Takako Morimoto, Hiroaki Oizumi, Katsuya Ohbuchi, Yuki Miyamoto, Junji Yamauchi

**Affiliations:** a Laboratory of Molecular Neuroscience and Neurology, Tokyo University of Pharmacy and Life Sciences, Hachioji, Tokyo 192-0392, Japan; b Tsumura Research Laboratories, Tsumura & Co., Inashiki, Ibaraki 200-1192, Japan; c Department of Pharmacology, National Research Institute for Child Health and Development, Setagaya, Tokyo 157-8535, Japan

Frontotemporal dementia (FTD) and amyotrophic lateral sclerosis (ALS) are neurodegenerative diseases that share overlapping genetic origins and similar cellular pathologies [1,2]. Expression of some FTD/ALS-associated mutated proteins affects glial cells as well as neuronal cells [3]. Thus, the clinical features are severe and often display developmental delay, spasticity, nystagmus, and optic atrophy.

Chromatin-modifying protein/charged multivesicular body protein 2B (CHMP2B) is a component of a protein metabolizing/degradation unit called endosomal sorting complex required for transport-III (ESCRT-III). CHMP2B is involved in the regulation of metabolizing cell surface proteins through the formation of multivesicular bodies (MVBs) [4,5]. The formation begins from a process in which misfolded and damaged proteins enter endosomes.

**Fig. 1:**
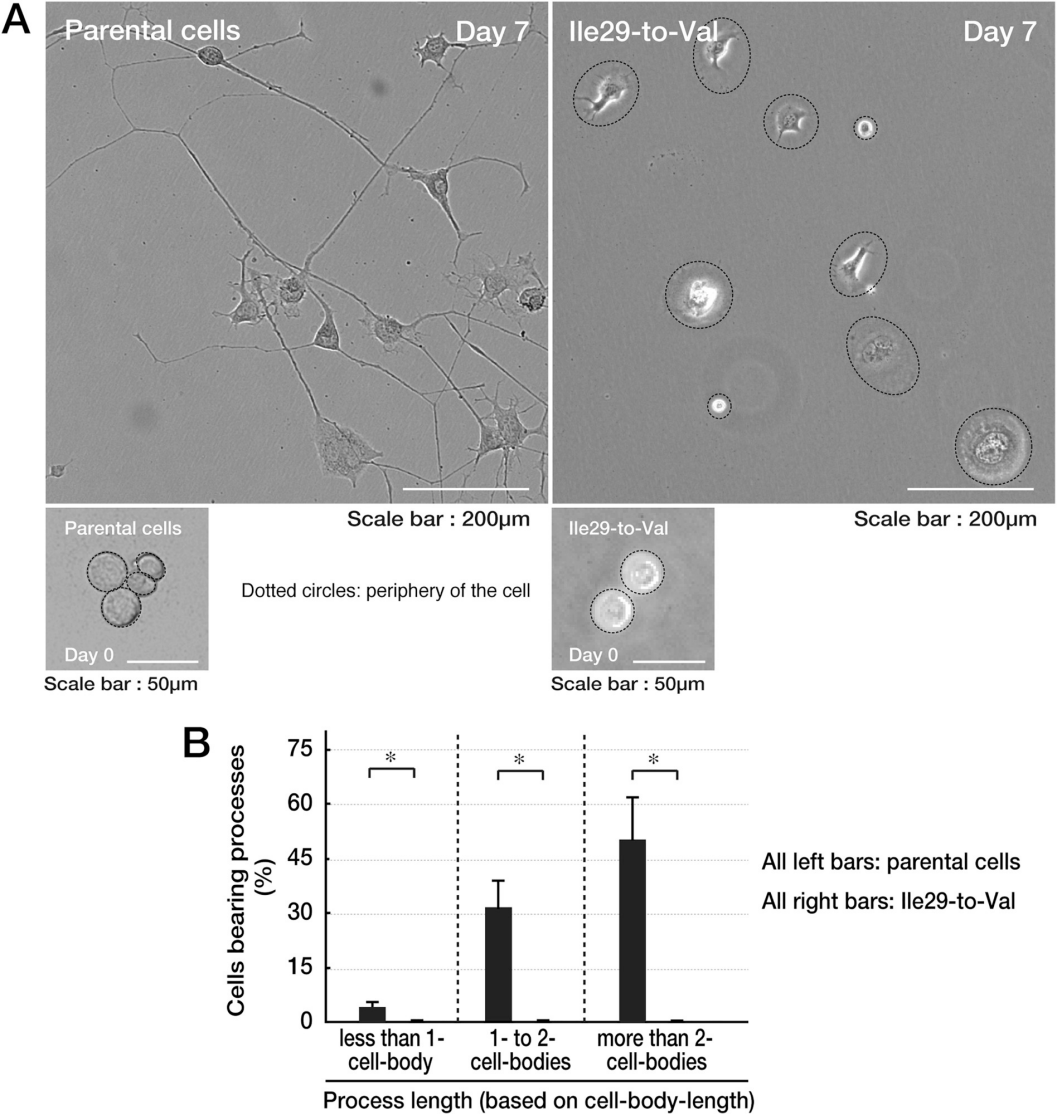
Cells harboring the mutant constructs of CHMP2B fail to exhibit differentiated phenotypes in N1E-115 cells. (A) Parental N1E-115 cells or cells harboring the mutant constructs were allowed to differentiate for 0 or 7 days. (B) After 7 days following the induction of differentiation, cells with more than one-cell-body-length of processes (indicated as 1- to 2-cell-bodies and >2-cell-bodies) from the cell bodies were considered differentiated phenotypes (*, p < .01 of Student's *t*-test; n = 3 fields). Cells harboring the mutant constructs failed to exhibit multiple processes.

**Fig. 2:**
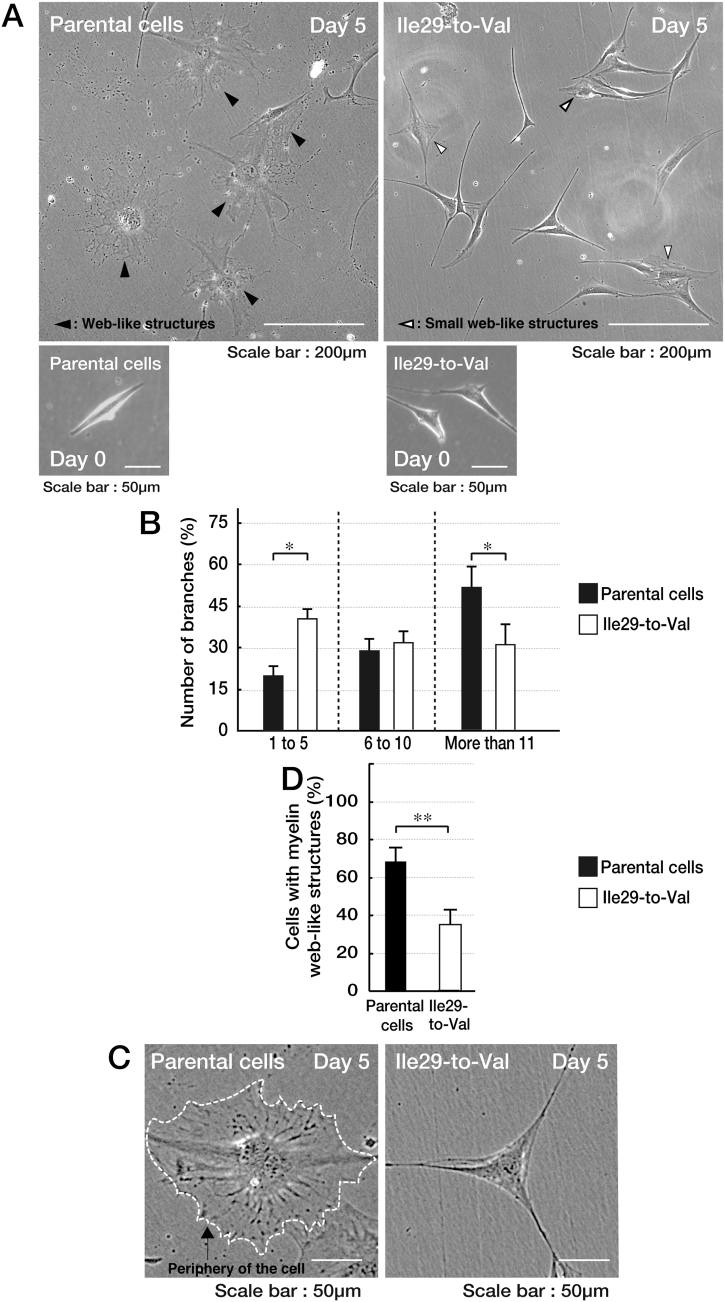
Cells harboring the mutant constructs of CHMP2B fail to exhibit differentiated phenotypes in FBD-102b cells. (A) Parental FBD-102b cells or cells harboring the mutant constructs were allowed to be differentiated for 0 or 5 days. (B) After 5 days following the induction of differentiation, cells with branches of 0 to 5, 5 to 10, or >11 from the cell bodies were counted. They are statistically shown (*, p < .01 of Student's *t*-test; n = 3 fields). Cells harboring the mutant constructs failed to exhibit multiple processes. (C) Parental FBD-102b cells or cells harboring the mutant constructs were allowed to be differentiated for 5 days. Representative image of a cell with myelin web-like structures is shown in the left panel, as compared to that of a cell without web-like structures in the right panel. (D) After 5 days following the induction of differentiation, cells with myelin web-like structures were counted. They are statistically shown (**, p < .05 of Student's *t*-test; n = 3 fields).

**Fig. S1:**
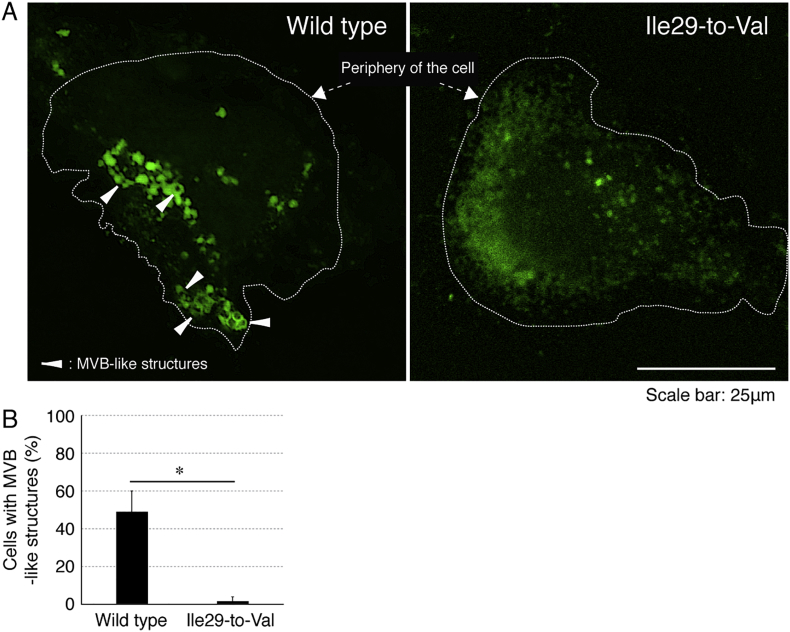
CHMP2B mutant proteins exhibit punctate structures whereas the wild type proteins form MVB-like structures. (A) COS-7 cells were transfected with the plasmid encoding the wild type CHMP2B or the mutant and obtained as fluorescence images (green). (B) Percentages of cells harboring MVB-like structures are statistically shown (*, p < .01 of Student's *t*-test; n = 3 fields).

**Fig. S2:**
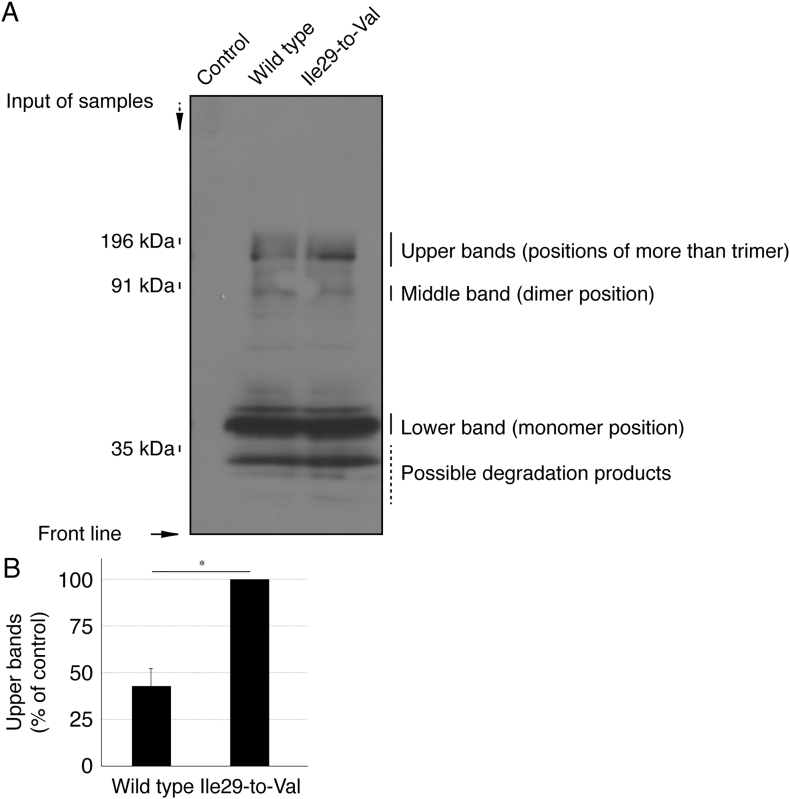
CHMP2B mutant proteins prefer to form polymeric structures in polyacrylamide electrophoresis. (A) The plasmid encoding the wild type or the mutant were transfected into COS-7 cells, subjected to non-denaturing polyacrylamide electrophoresis, and detected by immunoblotting. The predicted molecular mass ofCHMP2B is more than a 35 kDa marker band and indicated as a monomer position. (B) Protein bands corresponding to more than a trimer (possible protein aggregates) of CHMP2B proteins were densitometrically scanned and statistically calculated (*, p < .01 of Student's *t*-test; n = 3 bands).

**Fig. S3:**
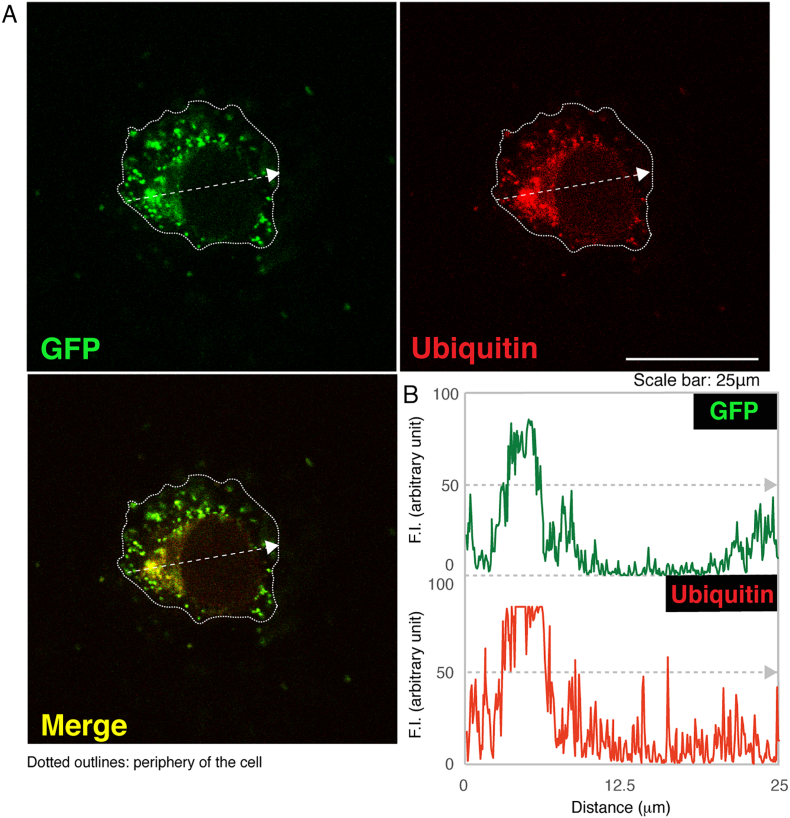
CHMP2B mutant proteins exhibit ubiquitin-positive punctate structures. (A) COS-7 were transfected with the plasmid encoding the CHMP2B mutant (green) and immunostained with an anti-ubiquitin antibody (red). Merged images (yellow) are also shown. (B) Fluorescence intensities (F.I., arbitrary unit) of green and red along dotted arrows in upper left and right images, respectively, are shown.

The Ile29-to-Val (I29V) mutation of CHMP2B is known to be associated with chromosome 3-linked familial FTD (FTD3) and ALS17 (OMIN Nos. 600795 and 600795, respectively) [[6], [7], [8], [9]]; however, the cellular and molecular mechanism underlying FTD3/ALS17 remains to be understood. Cells harboring CHMP2B mutant constructs failed to exhibit differentiated phenotypes with long processes in N1E-115 cells [10] as the neuronal cell model (Fig. 1). Similarly, cells harboring CHMP2B mutant constructs decreased processes with myelin web-like structures in FBD-102b cells [11] as the oligodendroglial cell model (Fig. 2). Also, wild type CHMP2B proteins formed MVB-like vesicular structures (Fig. S1) whereas mutant proteins did small protein aggregates (Fig. S2) and were mainly localized in ubiquitin-positive ones (Fig. S3) [12].

The Ile29-to-Val (I29V) mutation of CHMP2B is known to be associated with chromosome 3-linked familial FTD (FTD3) and ALS17 (OMIN Nos. 600795 and 600795, respectively) [[6], [7], [8], [9]]; however, the cellular and molecular mechanism underlying FTD3/ALS17 remains to be understood. Cells harboring CHMP2B mutant constructs failed to exhibit differentiated phenotypes with long processes in N1E-115 cells [10] as the neuronal cell model (Fig. 1). Similarly, cells harboring CHMP2B mutant constructs decreased processes with myelin web-like structures in FBD-102b cells [11] as the oligodendroglial cell model (Fig. 2). Also, wild type CHMP2B proteins formed MVB-like vesicular structures (Fig. S1) whereas mutant proteins did small protein aggregates (Fig. S2) and were mainly localized in ubiquitin-positive ones (Fig. S3) [12].

**Fig. S4:**
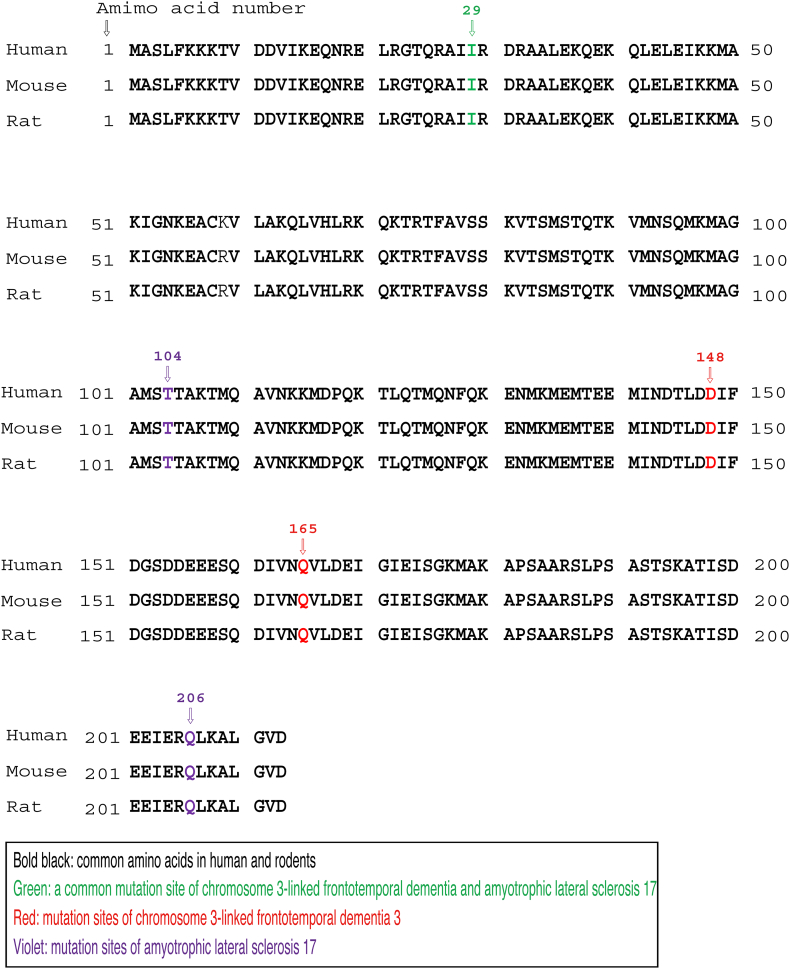
Comparison of primary sequences of CHMP2B in human and rodents. All CHMP2B proteins are composed of 210 amino acids. The Ile-29 position is present near the N-terminus.

**Fig. S5:**
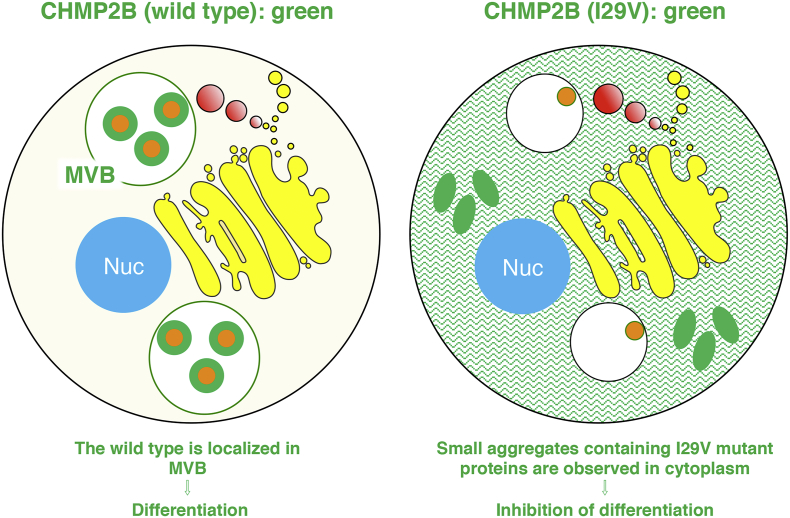
Schematic diagrams of cellular properties of wild type and FTD3/ALS17-associated mutant CHMP2B.

In amino acid sequences, the Ile-29 position in human and rodent CHMP2B is far from the positions of the respective mutations associated with FTD3 or ALS17 (Fig. S4). In addition to information as described above (Fig. S5), further studies on the relationship between FTD3 and/or ALS17 mutations and CHMP2B protein properties will allow us to understand how disease mutation causes cellular pathological effects.

In amino acid sequences, the Ile-29 position in human and rodent CHMP2B is far from the positions of the respective mutations associated with FTD3 or ALS17 (Fig. S4). In addition to information as described above (Fig. S5), further studies on the relationship between FTD3 and/or ALS17 mutations and CHMP2B protein properties will allow us to understand how disease mutation causes cellular pathological effects.

The following are the supplementary data related to this article.

Supplementary data to this article can be found online at https://doi.org/10.1016/j.ymgmr.2019.100458.
